# Moving rhythmically can facilitate naturalistic speech perception in a noisy environment

**DOI:** 10.1098/rspb.2025.0354

**Published:** 2025-04-09

**Authors:** Noémie te Rietmolen, Kristof Strijkers, Benjamin Morillon

**Affiliations:** ^1^ Institute for Language, Communication, and the Brain (ILCB), Aix-Marseille Université, Marseille, France; ^2^ Laboratoire Parole et Langage (LPL), Aix-Marseille Université & CNRS, Aix-en-Provence, France; ^3^ INSERM, Institut de Neurosciences des Systèmes (INS), Aix Marseille Université, Marseille, France

**Keywords:** speech perception, rhythm, audiomotor, synchronization, behaviour, speech-in-noise, auditory active sensing, action semantics, human psychophysics

## Abstract

The motor system is known to process temporal information, and moving rhythmically while listening to a melody can improve auditory processing. In three interrelated behavioural experiments, we demonstrate that this effect translates to speech processing. Motor priming improves the efficiency of subsequent naturalistic speech-in-noise processing under specific conditions. (i) Moving rhythmically at the lexical rate (~1.8 Hz) significantly improves subsequent speech processing compared to moving at other rates, such as the phrasal or syllabic rates. (ii) The impact of such rhythmic motor priming is not influenced by whether it is self-generated or triggered by an auditory beat. (iii) Overt lexical vocalization, regardless of its semantic content, also enhances the efficiency of subsequent speech processing. These findings provide evidence for the functional role of the motor system in processing the temporal dynamics of naturalistic speech.

## 1. Introduction

Listening to speech activates cortical regions of the sensorimotor system [1,2]. The functional role of these regions in speech processing and the conditions of their involvement are strongly debated [3–6]. Their role in linguistic processing has been highly investigated [5,7], but their putative role in the analysis of the temporal dynamics of the speech signal has been mostly overlooked [6]. However, studies on speech perception in adverse listening conditions—such as a noisy or multi-talker environment—show that tracking the temporal modulations of the speech signal of a speaker of interest facilitates speech comprehension [8,9]. Relatedly, the motor system is directly involved in the processing of auditory temporal information [10,11]. The motor system is the core amodal network supporting timing and time perception [12], enabling precise estimation of short durations [13,14]. It is also involved in beat and rhythm processing during passive listening [15,16]. Importantly, natural or precise human rhythmic movements occur around 0.8−2.5 Hz (~0.4–1.25 s) [17,18], which corresponds to our heightened sensitivity to music beats [19–22] and our capacity for auditory temporal attention [23,24]. Furthermore, beta (~20 Hz) activity, the default oscillatory mode in motor areas [25], is associated with the representation of temporal information [10,26–31]. Time estimation could thus rely on the neuronal recycling of action circuits and be implemented by internal simulation of movements [32,33].

Accordingly, a covert form of active sensing exists in the auditory domain, whereby motor sampling routines provide contextual information to optimize the parsing of sensory signals [11]. During perception of a melodic rhythmic stream, motor areas are directly involved in the processing of auditory temporal information [10,31]. Moreover, temporal segmentation of auditory information is improved when participants produce overt rhythmic movements compared to when they are staying still [10,23,34–37]. These studies provide evidence that the motor cortex administrates auditory temporal predictions and emphasize the fundamental role of motor brain areas and motor behaviour in providing contextual temporal information to sensory regions.

Whether these beneficial effects of motor behaviour for the temporal segmentation of auditory and musical input translate to speech and language processing is an open question. Speech and music have different dynamics [38] and speech is at best quasi-rhythmic, with no strict periodicity [39]. However, speech is also in essence a temporal signal, structured according to a hierarchy of time scales: In addition to fast varying phonetic feature dimensions, syllabic, lexical and phrasal slow modulations (1–7 Hz) are crucial to speech comprehension [40–43]. When speech is rhythmicized—with a rhythmically regular metrical prosodic structure—a musical prime that matches the metrical structure of the utterances (i.e. a rhythmically informative prime) has beneficial effects on speech and language processing. Such auditory informative rhythmic primes enhance the detection of target phonemes [44,45] and words [46] in the general adult population, as well as phonological production in hearing-impaired children [47]. Beyond auditory priming, motor activity also contributes to rhythmicized speech processing. The detection of target words improves when the metrical structure of the rhythmicized sentence is primed with an informative audiomotor rhythm [46] or when participants move in synchrony with the sentence’s rhythm [48,49]. Relatedly, producing periodic syllable sequences optimizes the discrimination of a subsequently presented syllable embedded in noise [50], and the individual rate of natural articulatory dynamics partly predicts speech comprehension performance of compressed sentences [51]. Critically, naturalistic (i.e. non-rhythmicized) sentences can also benefit from rhythmic priming. Generic periodic (i.e. temporally regular but non-informative) auditory primes improve syntax processing of sentences in healthy adults [52], typically developing children [53], and children with developmental language disorders [54–56]. However, the effect of non-informative motor rhythmic priming on naturalistic speech processing remains unexplored.

Building on the findings above, we sought to investigate whether (i) motor primes that are (ii) generic and periodic, and not informative about the specific metrical structure of the following sentence, could enhance (iii) comprehension of naturalistic speech-in-noise sentences. More concretely, we hypothesized that one contribution of motor areas to naturalistic speech processing concerns their implication in the analysis of some of the speech temporal dynamics, providing a contextual temporal frame to process linguistic information. In a series of behavioural experiments, we determined whether overt rhythmic motor activity (finger tapping) benefits subsequent speech comprehension. We also explored which motor rhythm has the most significant impact on speech processing and whether this effect is driven by internally generated motor behaviour rather than external sensorial information. Finally, we investigated whether more natural motor behaviour such as speech production would likewise aid subsequent speech comprehension. This is relevant because while the coupling of general motor movements (like tapping) to speech comprehension may be restricted to specific contexts, the motor behaviour of speech production is intrinsically linked to speech comprehension as both need to be continuously coordinated during our most frequent use of language, namely conversation [57]. Moreover, contrary to other motor behaviours, speech production, such as comprehension, contains linguistic information which may additionally recruit the motor cortex, notably through action-related words [58–62]. We thus investigated whether motor-to-auditory active sensing can be influenced above and beyond overt movement by the linguistic content of the produced word. In sum, the current study will investigate whether rhythmic motor activity aids the understanding of naturalistic speech, what factors drive that potential functional role of the motor cortex during comprehension, and if it also occurs for the ecological coupling between the production and perception of speech.

## 2. Methods

### (a) Participants

In total, 38, 47 and 45 French native participants (expriment 1: 5 male, average age = 23.7 years, s.d. = 3.64; experiment 2: 5 male, average age = 25.4 years, s.d. = 6.29; experiment 3: 7 male, average age = 25.6 years, s.d. = 6.31) were, respectively, recruited for experiments 1–3. 33 participants were involved in both experiments 2 and 3. The experiments followed the local ethics guidelines from Aix-Marseille University and were conducted in accordance with the Declaration of Helsinki. Informed consent was obtained from all participants before the experiments. All had (corrected to) normal audition and vision and reported no history of neurological, psychiatric or language-related disorders. Participants were not selected based on musical, dance or general audiomotor synchronization abilities, nor were they selected based on any other cognitive abilities.

### (b) Speech stimuli

The corpus consisted of 80 long French sentences that were spoken by a naive, native French speaker. To homogenize the signal-to-noise ratio (SNR) within and across sentences, all sentences were normalized in amplitude to 70 dB SPL and in pitch contour (mean: 205 Hz, s.d. = 37; mean variation from original files: 18%) using custom scripts in PRAAT [63]. Sentences lasted on average 5.5 s (s.d. = 0.75) and included minimally three accentual phrases. Phrases were manually extracted based on French accentual phrase boundary markers (i.e. a pre-boundary lengthening of the last syllable relative to the preceding syllable and/or an initial rise in fundamental frequency of the first syllable of the phrase [64,65]). Due to the high number of monosyllabic words in our sentences, and in order to separate the lexical and syllabic levels, the lexical level was defined as (extended) prosodic words [66,67], also called the clitic group [68] (i.e. content words combined with their subordinate function words). Phrasal, lexical and syllabic durations were on average 896 ms (s.d. = 475 ms), 553 ms (s.d. = 195 ms) and 199 ms (s.d. = 101 ms), respectively. On average, the lexical level contained 1.53 words (s.d. = 0.5) and 2.31 syllables (s.d. = 1.04).

Each sentence contained one target word (noun) that was semantically related to either the hand/arm effectors or the foot/leg effectors (20 target words in total; 10 for each effector (hand/arm: mean number of syllables 2.0 (s.d. = 0.63); foot/leg: mean number of syllables 2.4 (s.d. = 0.49); see electronic supplementary material); each repeated 4 times within the 80 sentences). For instance, the target word ‘heels’ refers to the foot effector in the following sentence: ‘Mary Tudor was the very first queen to wear heels made as high as possible’ (‘Mary Tudor était la toute première reine à vouloir porter des talons fabriqués aussi hauts que possible’). The position of the target within the sentences varied to ensure that participants listened attentively to the entire sentences. To ensure that participants could contribute to both experiments 2 and 3 (as described below) without encountering sentence repetition across experiments, we divided the stimuli into two lists, each containing 40 sentences and twice each target word.

Each sentence was masked with a speech-shaped noise (SSN), defined with the long-term average spectrum (LTAS) of each sentence, using custom scripts in PRAAT. The SSN spectrum was calculated with a fast Fourier transform after which a finite impulse response filter was convolved on a white noise with the length of the appropriate sentence. After mixing in the noise, stimuli were again amplitude-normalized to 70 dB.

### (c) Procedure

#### (i) Experiment 1

**Figure 1.**
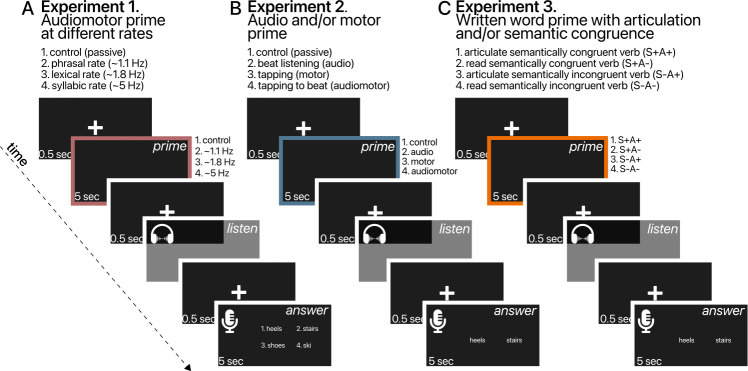
Schematic of an experimental trial for (A) experiment 1, (B) experiment 2 and (C) experiment 3. Each trial started with a prime after which participants heard a spoken sentence presented in noise. In the final stage of the trial, participants were asked to verbally identify which of several words presented on the screen they had heard in the sentence. Primes differed between the three experiments and corresponded to the different conditions (presented on the right). (A) passive (control), ~1.1, ~1.8 or ~5 Hz tapping for experiment 1. (B) passive (control), 1.7 Hz auditory beat (audio), rhythmic tapping (motor), or tapping to an auditory beat (audiomotor) for experiment 2. (C) articulation of a semantically congruent verb (S+A+), covert reading of a semantically congruent verb (S+A−), articulation of a semantically incongruent verb (S−A+), or covert reading of a semantically incongruent verb (S−A−).

This experiment occurred at the laboratory. Stimuli were presented in mono over headphones at a sampling rate of 44.1 kHz at 16 bits using Python 3.9 with the pyglet library on a Windows 10 platform. Volume was set at a comfortable level consistent across participants (~70 dB). Each participant was seated in a sound-attenuated room. Trials comprised three phases corresponding to prime, sentence and response (figure 1):

In phase I, participants were presented a white fixation cross (0.5 s) indicating the start of the trial, after which they heard an audible prime beat. They were instructed to, as quickly as possible, synchronize to the auditory beat by pressing the spacebar of the keyboard with their index finger. Periodic prime beats were presented for 5 s at either the phrasal (~1.1 Hz, s.d. = 0.15), lexical (~1.8 Hz, s.d. = 0.23) or syllabic (~5.0 Hz, s.d. = 0.33) rate of the upcoming sentence. In the control condition, no beat was presented and participants were instructed to remain passive. We asked participants to tap prior to (and not during) the sentence in order to avoid complications inherent to performing a dual task (i.e. tapping while attempting to understand a sentence embedded in noise).

Phase II started with the presentation of a white fixation cross (0.5 s), after which participants were presented with a spoken sentence embedded in noise. Participants were instructed to listen attentively to the sentence. The end of the sentence was indicated by a white fixation cross (0.5 s) after which, in phase III, four enumerated and written nouns appeared on the screen (figure 1). These nouns included the target word and three distractor words in the same effector-semantic category (i.e. four-alternative forced choice (4AFC) task). Participants were instructed to, as quickly and accurately as possible, indicate the word they had heard in the previous sentence by pronouncing the number next to it. The verbal response ensured that participants were not using an effector related to the target word (hand or foot; a control relevant for experiment 3; see below). Answers remained on the screen for 7 s, during which participants could provide their answer. The next trial followed automatically.

The experiment unfolded as follows. First, to ensure that participants understood the task requirements, the experiment began with a short practice session, consisting of eight trials that were similar to the experimental trials (i.e. two trials for each condition). Second, to adapt the noise level individually, participants performed a psychophysical staircase procedure, where the SNR was the varying parameter. The staircase consisted of 32 sentences recorded from the same French native speaker as the experimental stimuli and with similar durations. The staircase was set to obtain ~75% performance accuracy and took approximately 10 min to complete. Finally, the experiment proper (with trials consisting of phases I, II and III) consisted of 160 trials (40 per condition) divided into 4 blocks. Within each block, participants were presented with 40 sentences from a designated list (lists were randomized across participants), each paired with one of 4 possible primes (resulting in 10 trials per condition within each block). Repeating the sentences across the 4 blocks ensured that each prime-sentence (phases I–II) association was encountered once during the experiment. Within each block, conditions were presented in groups of 10 trials each (with a pseudo-randomized order across blocks and participants), aimed at minimizing confusion caused by the use of different primes across successive trials. Participants could take small breaks between blocks. The total duration of the experiment was approximately 1 h.

#### (ii) Experiments 2 and 3

Both experiments were run online using the FindingFive platform [69,70]. The procedure was similar to experiment 1 except for the type of prime in phase I (figure 1). In experiment 2, a 2-by-2 factorial design with listening and tapping as factors was employed. Participants would either (i) remain passive (control condition), (ii) listen to an auditory periodic beat presented at 1.7 Hz (audio), (iii) tap rhythmically at their preferred rate (motor) or (iv) tap in synchrony to a beat presented at 1.7 Hz (audiomotor). In experiment 3, a 2-by-2 factorial design with articulation and semantic congruency as factors was employed. Participants would either (i) articulate a written verb, an action-related word semantically congruent with the target noun in the upcoming sentence (S+A+; e.g. utter ‘kick’ when the upcoming noun is ‘heels’; see electronic supplementary material), (ii) covertly read a semantically congruent verb (S+A−; read ‘kick’ when the upcoming noun is ‘heels’), (iii) articulate a semantically incongruent verb (S−A+; e.g. utter ‘grab’ when the upcoming noun is ‘heels’), or (iv) covertly read a semantically incongruent verb (S−A−; e.g. read ‘grab’ when the upcoming noun is ‘heels’).

Due to the complexity of task instructions and a high error rate observed in a pilot study for these two online experiments, we simplified the procedure by: (i) keeping the practice session but removing the staircase procedure and directly (ii) setting the noise levels to+2 dB in phase II of the main experiment trials; and, in phase III of the trials, (iii) presenting only two (instead of four) semantically related words (the target and one distractor, 2AFC task), (iv) asking participants to pronounce the word (instead of the number) that they recognized as the target, and (v) allowing 5 s to provide their answer. The total duration of these online experiments was approximately 45 min.

#### (iii) Data analyses

The behavioural data were analysed in R [71] with linear mixed-effects models, using the lme4 [72] and emmeans [73] packages. We estimated per condition three variables: (i) the comprehension accuracy (proportion of correct responses, as a percentage), (ii) the response speed in correct trials (correct reaction times (RTs), log-transformed, in ms) and (iii) our main variable of interest, the inverse efficiency score (IES), which combines the effects of speed and accuracy into a summary statistic (correct RT (log)/proportion correct) [74]. This choice was informed by prior research using rhythmic primes, which has often variably reported behavioural benefits in RTs [44,45] or accuracy [52,54,56]. IES controls for potential speed/accuracy trade-offs and indexes the overall (inverse) efficiency of the participant’s responses. However, for the sake of transparency, we also report on the accuracy and correct RT results.

Participants were excluded from the study if they did not perform significantly above chance in any of the conditions (i.e. 38%, 63%, 63% for experiments 1−3, respectively; one-tailed binomial test, α = 0.05), as this indicated that the task was excessively difficult. In total, 3, 6 and 7 participants were excluded from experiments 1, 2 and 3, respectively, resulting in final participant counts of 35, 41 and 38 for each experiment. Responses with a RT < 0.2 s (1, 3, 0 for experiments 1−3 [75]) and missed trials (4.1%, 4.8%, 1.7% for experiments 1−3) were excluded from the analysis.

Finally, we estimated the tapping regularity of participants, in experiments 1 and 2. Outlier taps were identified at the subject level using the interquartile range (IQR), excluding intertap intervals smaller or greater than 3 times the IQR below and above the first and third quartile, respectively. Then, for each trial, we computed the mean and standard deviation of the inter-tap intervals. We then derived the coefficient of variation (CV), expressed as the relative standard deviation, i.e. the ratio between the standard deviation of the inter-tap intervals and the tempo. All trials with fewer than 3 taps were excluded from this analysis. In total, 11 participants were excluded from this tapping analysis for experiment 2, due to the insufficient quality of online tapping recordings. Produced articulations were not recorded in experiment 3.

We used linear mixed models to investigate how well our different conditions predicted our three variables of interest. We included participants, sentence repetition index (from 1 to 4; see §2) and stimuli as random variables. More specifically, intercepts for participants, repetition index and stimuli, as well as by-stimuli random slopes for the effects of the experimental conditions best accounted for the underlying random variability. Given the high number of observations (>300), the normality and homoscedasticity of the residuals were assessed through visual inspection of histograms and QQ-plots, and by evaluating the absolute values of skewness and kurtosis. Residuals were found to respect homoscedasticity and be approximately normal: all residuals had skewness in the [−2, 2] range and kurtosis in the [−7, 7] range [76]. Finally, we computed multiple pairwise comparisons between the conditions of each experiment using estimated marginal means, applying Tukey’s correction to control for Type I errors while maintaining statistical power across comparisons.

## 3. Results

### (a) Experiment 1

In the first experiment, we set out to determine whether overt motor activity (i.e. rhythmic finger tapping) benefits speech comprehension. The main acoustic temporal modulation in naturalistic speech occurs at ~5 Hz and approximates the syllabic rate [38,43,77]. However, natural motor dynamics occur at a lower scale (∼0.8−2.5 Hz) [18,19,32], a range encompassing lexical and prosodic phrasal rates in naturalistic speech [42]. We hence jointly investigated whether overt rhythmic movements at either the phrasal, lexical, or syllabic level positively impact speech comprehension. Moreover, we relied on the assumptions that motor activity optimally contributes to speech perception when the speech SNR is low [78] and the speech rate is natural [43].

**Figure 2.**
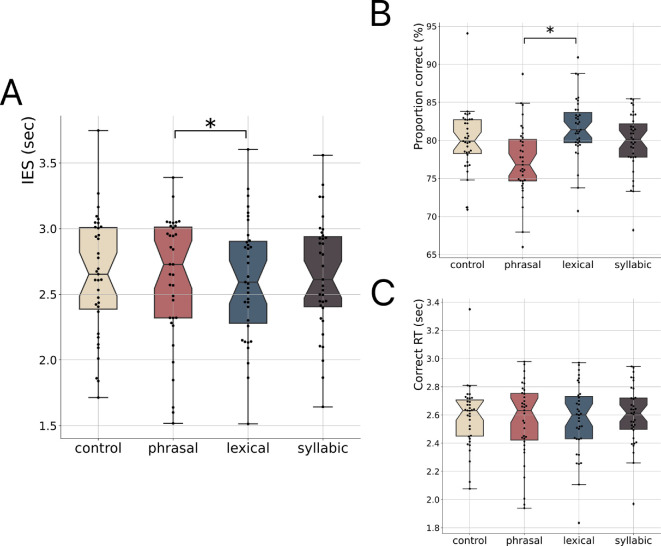
Experiment 1. (A) Inverse efficiency scores (IES), (B) proportion correct, and (C) correct reaction times (RT) for the no tapping (control, in beige), tapping at ~1.1 Hz (phrasal rate, in red), tapping at ~1.8 Hz (lexical rate, in blue), and tapping at ~5 Hz (syllabic rate, in brown) conditions. Black dots indicate individual participants. Boxplots represent median and 1.5 times the interquartile range (*n* = 35; *p < 0.05, Tukey-corrected).

Participants listened to long naturalistic French sentences (see §2), preceded by a priming phase where they either remained passive (control condition) or tapped in phase with an auditory beat at phrasal (1.1 Hz), lexical (1.8 Hz) or syllabic (5.0 Hz) rates. Inter-tap intervals were 0.92 s (s.d. = 0.14; CV: mean = 11.46%, s.d. = 0.09) for the phrasal rate, 0.47 s (s.d. = 0.06; CV: mean = 11.42%, s.d. = 0.06) for the lexical rate, and 0.21 s (s.d. = 0.03; CV: mean = 12.66%, s.d. = 0.08) for the syllabic rate. We examined the pairwise comparisons between our conditions for IESs, comprehension accuracy (proportion correct) and response speed (correct RTs (figure 2; electronic supplementary material, table S1 and figure S1). We observed a significant benefit of tapping at the lexical (~1.8 Hz) rate on both IES and proportion correct metrics, but not on correct RT. Specifically, IES was significantly smaller (i.e. more efficient) in the lexical than in the phrasal rate condition (~1.1 Hz; *b* = −0.06, s.e. = 0.02, *p* = 0.029, *n* = 35, Tukey-corrected for multiple comparisons). Similarly, comprehension accuracy was significantly higher in the lexical than phrasal rate condition (*b* = 0.04, s.e. = 0.01, *p* = 0.016).

These results indicate that finger tapping at the lexical rate (~1.8 Hz) significantly facilitates speech processing more than tapping at another rate (phrasal). However, no direct improvement compared to the passive (nor syllabic) control condition was observed. We reasoned that changing the rate of the rhythmic prime across trials could have confused participants, making it difficult to benefit from an overt motor strategy compared to the ubiquitous passive audio condition. Moreover, in this first experiment, movements occurred in phase with an auditory beat. Hence, the observed effects could be due to an overt motor implication and/or the influence of the externally triggered auditory prime.

### (b) Experiment 2

In a second experiment, we hence investigated whether an auditory and/or motor rhythmic prime—at a fixed rate—enhances naturalistic speech processing. Beyond the advantages observed in motor-related studies [46,48,49], prior research in the general adult population has demonstrated behavioural benefits resulting from an auditory rhythmic prime, whether informative [44,46,47] or non-informative [52]. To investigate whether a spontaneous (non-informed) motor prime can influence naturalistic speech processing, we employed a 2-by-2 factorial design with listening and tapping as factors, in a 2AFC version of the same speech-in-noise comprehension task (see §2). Each sentence was preceded by a 5 s long priming phase, in which participants either remained passive (control condition), listened to an auditory beat at 1.7 Hz (audio), moved rhythmically at their own rate (motor), or tapped in phase with an auditory beat at 1.7 Hz (audiomotor). Sound streams were presented at the lexical (~1.7 Hz) rate of the upcoming sentence, in accordance with the results of experiment 1, and spontaneous tapping also occurs naturally around 1.7 Hz [18,19,32].

Accordingly, inter-tap intervals in the motor-only condition were 0.52 s (s.d. = 0.22; CV: mean = 20.4%, s.d. = 0.8). We first examined the main effects of the listening and tapping factors on IES, comprehension accuracy and correct RT. The linear mixed model analysis (*n* = 41) revealed a significant main effect of tapping on IES (*b* = −0.04, *p* = 0.001), comprehension accuracy (*b* = 0.02, *p* = 0.019) and correct RT (b = −0.03, *p* = 0.008; table 1). The listening factor had a main effect on comprehension accuracy (*b* = −0.04, *p* < 0.001), correct RT (b = 0.02, *p* = 0.034), but not on IES. No significant interactions were observed.

**Table 1.** Experiment 2. Summary of the statistical results for the main effects of listening and tapping factors in the two-by-two design analysis, using linear mixed models (*n* = 41). Results are reported for inverse efficiency scores (IES), comprehension accuracy (proportion correct as a percentage) and response speed (correct reaction times, RT). Significant *p*-values are in bold.

	IES		% correct		correct RT	
contrast	b	*p*	b	*p*	b	*p*
C(listening)[T.1]	0.0105	0.4179	0.0371	**0.0003**	0.0247	**0.0341**
C(tapping)[T.1]	−0.0422	**0.0011**	0.0237	**0.0193**	−0.0309	**0.0079**
C(listening)[T.1]:C(tapping)[T.1]	0.0054	0.7659	−0.0213	0.1376	−0.0018	0.9116

**Figure 3.**
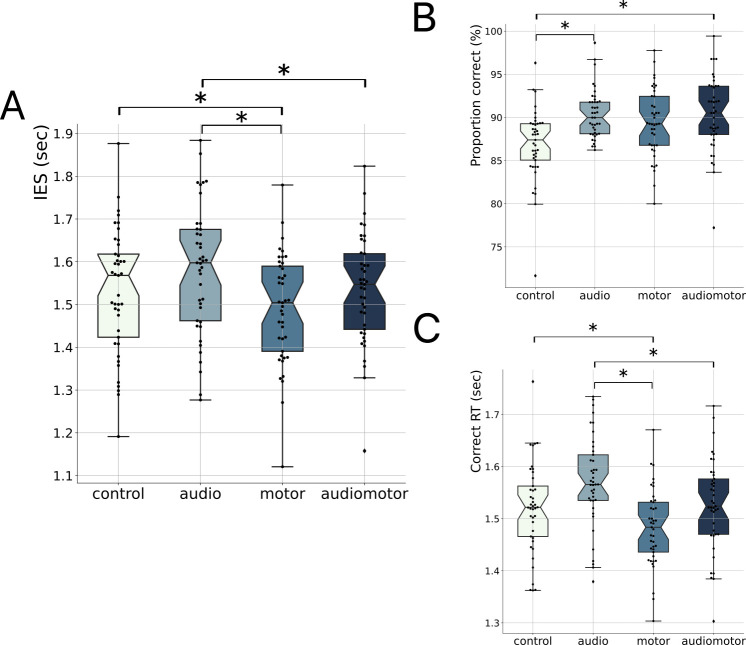
Experiment 2. (A) Inverse efficiency scores (IES), (B) proportion correct and (C) correct reaction times (RT) for the passive (control, in beige), audio (in light blue), motor (in medium blue) and audiomotor synchronization (in dark blue) conditions. Same conventions as in figure 1 (*n* = 41; *p < 0.05, Tukey-corrected).

Pairwise comparisons (figure 3; electronic supplementary material, table S2 and figure S2; Tukey-corrected for multiple comparisons) further showed that IES was significantly smaller in the motor condition compared to the audio (*b* = 0.05, s.e. = 0.01, *p* < 0.001) and the control (*b* = 0.04, s.e. = 0.01, *p* = 0.006) conditions, and in the audiomotor condition compared to the audio condition (b = 0.04, s.e. = 0.01, *p* = 0.021). Comprehension accuracy was significantly higher in the audiomotor (*b* = 0.04, s.e. = 0.01, *p* < 0.001) and audio conditions (*b* = 0.04, s.e. = 0.01, *p* = 0.002) compared to the control condition. Finally, correct RT was significantly shorter in the motor condition compared to the audio (*b* = 0.06, s.e. = 0.01, *p* < 0.001) and control conditions (*b* = 0.03, s.e. = 0.01, *p* = 0.039), and in the audiomotor condition compared to the audio condition (*b* = 0.03, s.e. = 0.01, *p* = 0.022).

These results show that a rhythmic motor prime at the lexical (~1.8 Hz) rate significantly facilitates speech-in-noise processing, and that this positive impact of motor priming is not influenced by whether it is self-generated or triggered by an auditory beat.

### (c) Experiment 3

In a final experiment, we investigated whether the observed facilitatory effect of motor tapping generalizes to a more natural motor prime in the context of speech processing, namely, the articulation of a contextually relevant word. This is relevant since speech production and perception naturally alternate—and hence prime each other—during ecological conversations. Moreover, as with most motor behaviours, rhythmicity is observed in the articulatory domain [43]. We tested whether the overt articulation of the prime and/or effector-specificity at the semantic level between the prime and the sentence enhances speech comprehension just as it did for motor tapping.

To this end, we employed a 2-by-2 factorial design with articulation and semantic congruency as factors. Participants either covertly read or overtly articulated (A− or A+) a lexical prime, an action-related word that was either semantically congruent or incongruent (S+ or S−) with the target word in the sentence (see §2 and electronic supplementary material). We first examined the main effects of the articulation and semantic congruency factors. The linear mixed model analysis (*n* = 38) revealed a significant main effect of articulation on IES (*b* = −0.03, *p* = 0.034) and correct RT (*b* = −0.03, *p* = 0.038), but not on comprehension accuracy (table 2). No significant main effects of semantic congruency and no significant interactions were observed.

**Table 2.** Experiment 3. Summary of the statistical results for the main effects of semantic congruency and articulation factors in the two-by-two design analysis, using linear mixed models (*n* = 38). Same conventions as in table 1.

	IES		% correct		correct RT	
contrast	b	*p*	b	*p*	b	*p*
C(congruency)[T.1]	−0.008	0.5873	−0.007	0.5352	−0.0043	0.7401
C(articulation)[T.1]	−0.0311	**0.0344**	−0.0031	0.7867	−0.0268	**0.038**
C(congruency)[T.1]:C(articulation)[T.1]	−0.0088	0.6724	0.0179	0.2642	−0.0046	0.801

**Figure 4.**
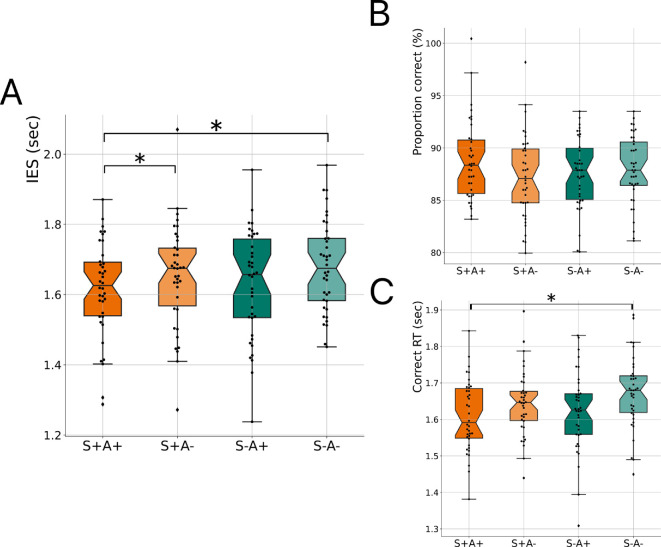
Experiment 3. (A) Inverse efficiency scores (IES), (B) proportion correct, and (C) correct reaction times (RT) for the articulation of a semantically congruent verb (S+A+, in dark orange), the covert reading of a semantically congruent verb (S+A−, in light orange), the articulation of a semantically incongruent verb (S−A+, in dark green), and the covert reading of a semantically incongruent verb (S−A−, in light green) conditions. Same conventions as in figure 1 (*n* = 38; *p < 0.05, Tukey-corrected).

Pairwise comparisons (figure 4; electronic supplementary material, table S3; figure S3; Tukey-corrected for multiple comparisons) further showed that IES was significantly smaller in the overt congruent condition (S+A+) than in both the covert congruent (S+A−; *b* = −0.09, s.e. = 0.01, *p* = 0.034) and covert incongruent condition (S−A−; *b* = −0.05, s.e. = 0.01, *p* = 0.006). Comprehension accuracy was not significantly different between conditions. Finally, correct RT was significantly lower in the overt congruent condition (S+A+) compared to the covert incongruent condition (S−A−; *b* = −0.04, s.e. = 0.01, *p* = 0.029).

These results show that overt lexical articulation—independently of its semantic content—improves the efficiency of speech-in-noise processing.

## 4. Discussion

The present study explored the role of motor activity in naturalistic speech-in-noise comprehension, building on the theory that the motor system is not merely an executor of movements but actively contributes to the integration and reuse of temporal information [79–82]. Through a series of three interrelated behavioural experiments, we investigated (i) whether generic periodic primes (i.e. temporally regular but non-informative) can facilitate naturalistic (i.e. non-rhythmicized) speech-in-noise comprehension, (ii) whether this effect is rate-specific (experiment 1), (iii) independent of auditory rhythmic stimulation (experiment 2) and (iv) whether it generalizes to more natural rhythmic movements involved in speech production (articulation) and/or to covert motor activation through action-related verbs (experiment 3). Our results mainly indicate that: (i) a rhythmic motor prime at the lexical (defined as prosodic words; clitic groups [66–68]) rate (~1.5−2 Hz) significantly facilitates naturalistic speech-in-noise processing, and more than tapping at other rates (phrasal or syllabic); (ii) the positive impact of motor priming is not influenced by whether it is self-generated or triggered by an auditory beat; and (iii) overt lexical articulation improves the efficiency of speech-in-noise processing.

Collectively, these experiments underscore the interwoven relationship between action and perception, expanding our understanding of the motor system’s involvement in language comprehension. They generalize prior work obtained on rhythmicized speech to unpredictable, naturalistic speech. These earlier investigations highlighted the influence of an informative rhythmic prime, whether auditory and/or motor, which corresponded to the prosodic structure of subsequent rhythmicized sentences [44–47], as well as movements that were temporally aligned with the sentence’s rhythm [48,49]. These former studies were often interpreted in the framework of the dynamic attending theory (DAT) [83–85]: The regularity in the prime was informative about the strong metrical structure of the subsequent rhythmicized sentence, providing predictable temporal cues that facilitated directing attention to salient moments of the speech stream [10,86]. In contrast, our study employed generic periodic (i.e. temporally regular) primes (auditory and/or motor) that did not convey information about the specific structure of the subsequent naturalistic (i.e. non-rhythmicized) sentences. They generalize to the motor modality—and to sentences comprehension—prior observations that periodic auditory primes improves syntactic processing of naturalistic sentences [52–56]. That generic motor primes enhance speech-in-noise comprehension not only extends prior findings but also suggests a pre-activation of general auditory attention, potentially involving the auditory dorsal pathway—which connects motor and auditory cortices—in comprehension processes. Future neuroimaging studies are needed to test this hypothesis and further elucidate the neural mechanisms underlying these behavioural effects.

The beneficial effects of auditory periodic primes on subsequent speech processing were previously investigated at a frequency of 2 Hz [52–56]. However, speech tracking can occur at multiple linguistic time scales: at the syllabic level, approximated by the speech rate [38,87]; at the word or lexical stress level [88]; and at higher-order prosodic levels, such as multiword intonational units [89,90]. Here we show that the beneficial effect of a generic periodic prime on speech comprehension is strongest at a frequency of approximately 1.5−2 Hz. This observed rate-selectivity at the lexical rate rules out the possibility that the facilitation is driven by nonspecific arousal or motor preparation effects. It also challenges assumptions about the role of the motor system in syllable segmentation [43,91] and suggests a contribution to prosodic word segmentation. Our findings connect with prior research on the role of cross-modal contributions to word segmentation, including visual [92,93] and somatosensory [94] modalities. This result is especially striking considering the language under investigation in the current study, French. French is a syllable-timed language [95,96] with post-lexical stress (i.e. at the level of the phrase [97,98]). As such, French features a prosodic system that is more inclined to favour syllable-based segmentation (e.g. [99–101]). If the preferred rate of the prime depended on the rhythmic structure of the studied language, the French prosodic system would predict a preference for syllabic (metrical unit) or phrasal (domain of stress) segmentation. Instead, our results show that speech-in-noise comprehension benefits specifically from tapping at the lexical-level time scale. This cannot be easily interpreted as being related to the rhythmic prosodic structure of the French language and is more easily explained by a universal segmentation strategy. This suggests that our effect is not language-dependent and should generalize across languages. Instead, this preferred tapping rate is in line with what is observed in music research. That is, ~1.5−2 Hz closely approximates the optimal rate for motor rhythmic precision [17,18,23], but acutely also of auditory temporal attention. It defines our heightened sensitivity to music beats [19–22], our auditory temporal attention capacity [23,24], and seems to characterize the neurophysiology of the auditory dorsal pathway, which links auditory and motor cortices [31]. That such music results translate to speech processing, with a preferred tempo of around 1.5−2 Hz, suggests the domain-generality of the motor contribution to auditory processing—encompassing both speech and music domains—and a natural inclination for motor-driven periodic processing at this frequency. This is also in line with studies showing that auditory temporal attention is optimal for ~1.5−2 Hz periodic rhythms, regardless of whether they are composed of linguistic or non-linguistic stimuli [24] and that rhythm discrimination capacity is unaffected by both stimulus type and the listener’s native language [102]. Ultimately, the question of the origin of the rate-specific motor contribution to speech processing remains to be conclusively addressed.

The rate-selectivity of the motor contribution to speech processing is indeed currently a topic of debate, with evidence suggesting either a preference for slow rates (~1.5−2 Hz, corresponding to phrasal or lexical rate), fast rates (~4.5 Hz, syllabic rate) or no selectivity. Notably, audiomotor synchronization demonstrates higher accuracy at ~2 Hz than at 4.5 Hz, regardless of the motor effector (hand/mouth) involved [103]. In speech-in-noise comprehension, neural tracking for correctly comprehended trials is more pronounced in the left premotor cortex at the phrasal rate (~1 Hz) and in the left middle temporal cortex at the word rate (~2 Hz [8]). Conversely, the individual fast (~4.5 Hz) rate of natural articulatory dynamics partially predicts speech comprehension performance for compressed sentences [51]. Finally, producing periodic syllable sequences enhances the discrimination of subsequently presented syllables embedded in noise, an effect observed at both 2 and 4.5 Hz [50].

The current study also demonstrated that the benefits of overt finger tapping extend to speech production (i.e. articulation). This result is compatible with conclusions that speech perception engages articulatory action [104] and that auditory-motor synchronization is stable across effectors (hand/mouth), which supports the hypothesis of a central clock mechanism subserving the different articulators [105]. Critically, it expands the role of the motor system for segmenting speech input to everyday language use. That is, language in dialogue is the most frequent and functional form of language processing (e.g. [106]). Given that in dialogue and conversation, there is a continuous interaction between the production and perception of language, the current demonstration that single-word articulation aids subsequent comprehension may therefore suggest that motor-to-auditory active sensing is a process that naturally occurs in our everyday interactions with others. Put differently, while the tapping results experimentally highlight the role of the motor system during comprehension, the articulation data expands that role to normal human communication in general. Intriguingly, the semantic content of the prime had no significant impact on the articulatory effect. While there may be numerous reasons behind this absence, our data does not support a complementary effect of meaning upon active sensing.

Regarding limitations, as speech was always presented in noise, it is unclear whether motor activity enhances comprehension *per se* or optimizes the allocation of attentional resources to discriminate speech from noise [78,107]. In the present work, we avoided potential dual-task issues by asking participants to move prior to listening to noisy sentences [108]. Whether ongoing movements *during* naturalistic speech-in-noise listening also facilitate comprehension or not is an open issue. Another limitation is that this study did not assess participants' musical or dance training background, preventing us from determining whether the observed benefits of motor activity on speech-in-noise comprehension vary across populations with different levels of rhythmic or musical experience [109–112]. As such, it remains unclear whether our results are more pronounced in musicians or individuals with strong audiomotor synchronization abilities. Future research could examine whether moving is particularly advantageous for individuals with strong rhythmic skills and whether these benefits are similarly constrained by rate limitations. Relatedly, recent studies have pointed at a bimodal distribution in the general population, wherein individuals naturally either align well to speech rhythms or not (high/low synchronizers [50,113,114]. In the context of rhythmicized sentences, while a rhythmic motor prime alone does not globally improve comprehension, the regularity of motor tapping and whether participants are low or high synchronizers modulates performance accuracy [46]. This further suggests a potential link between enhanced comprehension and the activation of the auditory dorsal pathway through periodic (auditory and/or motor) primes [86]. Whether low and high synchronizers, who differ in a number of behaviours, benefit differently from an overt motor strategy to comprehend naturalistic speech remains to be investigated. Finally, in experiment 3 we could not register the produced articulations, which typically occur between 2 and 8 Hz [43,115]. Whether articulatory rate or regularity impacts subsequent speech comprehension is an open question that remains to be addressed.

In conclusion, the findings underscore the intertwining of action and perception in language comprehension, shedding light on the active role of the motor system for naturalistic speech-in-noise processing.

## Data Availability

Data and analysis scripts are available on GitHub [116]. Supplementary material is available online [117].

## References

[1] Hauk O, Johnsrude I, Pulvermüller F. 2004 Somatotopic representation of action words in human motor and premotor cortex. Neuron **41**, 301–307. (10.1016/s0896-6273(03)00838-9) 14741110

[2] Wilson SM, Saygin AP, Sereno MI, Iacoboni M. 2004 Listening to speech activates motor areas involved in speech production. Nat. Neurosci. **7**, 701–702. (10.1038/nn1263) 15184903

[3] Hickok G, Houde J, Rong F. 2011 Sensorimotor integration in speech processing: computational basis and neural organization. Neuron **69**, 407–422. (10.1016/j.neuron.2011.01.019) 21315253 PMC3057382

[4] Kotz SA, Schwartze M. 2010 Cortical speech processing unplugged: a timely subcortico-cortical framework. Trends Cogn. Sci. **14**, 392–399. (10.1016/j.tics.2010.06.005) 20655802

[5] Schomers MR, Pulvermüller F. 2016 Is the sensorimotor cortex relevant for speech perception and understanding? An integrative review. Front. Hum. Neurosci. **10**, 435. (10.3389/fnhum.2016.00435) 27708566 PMC5030253

[6] Scott SK, McGettigan C, Eisner F. 2009 A little more conversation, a little less action—candidate roles for the motor cortex in speech perception. Nat. Rev. Neurosci. **10**, 295–302. (10.1038/nrn2603) 19277052 PMC4238059

[7] Skipper JI, Devlin JT, Lametti DR. 2017 The hearing ear is always found close to the speaking tongue: review of the role of the motor system in speech perception. Brain Lang. **164**, 77–105. (10.1016/j.bandl.2016.10.004) 27821280

[8] Keitel A, Gross J, Kayser C. 2018 Perceptually relevant speech tracking in auditory and motor cortex reflects distinct linguistic features. PLoS Biol. **16**, e2004473. (10.1371/journal.pbio.2004473) 29529019 PMC5864086

[9] Zion Golumbic EM *et al*. 2013 Mechanisms underlying selective neuronal tracking of attended speech at a ‘cocktail party’. Neuron **77**, 980–991. (10.1016/j.neuron.2012.12.037) 23473326 PMC3891478

[10] Morillon B, Baillet S. 2017 Motor origin of temporal predictions in auditory attention. Proc. Natl Acad. Sci. USA **114**, E8913–E8921. (10.1073/pnas.1705373114) 28973923 PMC5651745

[11] Morillon B, Hackett TA, Kajikawa Y, Schroeder CE. 2015 Predictive motor control of sensory dynamics in auditory active sensing. Curr. Opin. Neurobiol. **31**, 230–238. (10.1016/j.conb.2014.12.005) 25594376 PMC4898262

[12] Merchant H, Yarrow K. 2016 How the motor system both encodes and influences our sense of time. Curr. Opin. Behav. Sci. **8**, 22–27. (10.1016/j.cobeha.2016.01.006)

[13] Coull JT, Vidal F, Nazarian B, Macar F. 2004 Functional anatomy of the attentional modulation of time estimation. Science **303**, 1506–1508. (10.1126/science.1091573) 15001776

[14] Morillon B, Kell CA, Giraud AL. 2009 Three stages and four neural systems in time estimation. J. Neurosci. **29**, 14803–14811. (10.1523/jneurosci.3222-09.2009) 19940175 PMC6666014

[15] Chen JL, Penhune VB, Zatorre RJ. 2008 Listening to musical rhythms recruits motor regions of the brain. Cereb. Cortex **18**, 2844–2854. (10.1093/cercor/bhn042) 18388350

[16] Grahn JA, Rowe JB. 2013 Finding and feeling the musical beat: striatal dissociations between detection and prediction of regularity. Cereb. Cortex **23**, 913–921. (10.1093/cercor/bhs083) 22499797 PMC3593578

[17] MacDougall HG, Moore ST. 2005 Marching to the beat of the same drummer: the spontaneous tempo of human locomotion. J. Appl. Physiol. **99**, 1164–1173. (10.1152/japplphysiol.00138.2005) 15890757

[18] Repp BH, Su YH. 2013 Sensorimotor synchronization: a review of recent research (2006–2012). Psychon. Bull. Rev. **20**, 403–452. (10.3758/s13423-012-0371-2) 23397235

[19] Fraisse P, Deutsch D. 1982 Rhythm and tempo. In Psychology of language: cognition and perception, pp. 149–180. Amsterdam, The Netherlands: Elsevier. (10.1016/B978-0-12-213562-0.50010-3)

[20] London J. 2012 Hearing in time: psychological aspects of musical meter. Oxford, UK: Oxford University Press.

[21] McAuley JD. 2010 Tempo and rhythm. In Music perception (eds M Riess Jones, RR Fay, AN Popper), pp. 165–199. New York, NY: Springer.

[22] Moelants D. 2002 Preferred tempo reconsidered (eds C Stevens, D Burnham, G McPherson, E Schubert, J Renwick). In Proceedings of the 7th International Conference on Music Perception and Cognition, Sydney, Australia. Causal Productions.

[23] Zalta A, Petkoski S, Morillon B. 2020 Natural rhythms of periodic temporal attention. Nat. Commun. **11**, 1051. (10.1038/s41467-020-14888-8) 32103014 PMC7044316

[24] Gupta A, Matthews TE, Penhune VB, Morillon B. 2024 Behavioral evidence for two modes of attention. BioRxiv. (10.1101/2024.09.12.612641)

[25] Murthy VN, Fetz EE. 1992 Coherent 25- to 35-Hz oscillations in the sensorimotor cortex of awake behaving monkeys. Proc. Natl Acad. Sci. USA **89**, 5670–5674. (10.1073/pnas.89.12.5670) 1608977 PMC49354

[26] Arnal LH, Doelling KB, Poeppel D. 2015 Delta–beta coupled oscillations underlie temporal prediction accuracy. Cereb. Cortex **25**, 3077–3085. (10.1093/cercor/bhu103) 24846147 PMC4537446

[27] Fujioka T, Trainor LJ, Large EW, Ross B. 2009 Beta and gamma rhythms in human auditory cortex during musical beat processing. Ann. NY. Acad. Sci. **1169**, 89–92. (10.1111/j.1749-6632.2009.04779.x) 19673759

[28] Iversen JR, Repp BH, Patel AD. 2009 Top-down control of rhythm perception modulates early auditory responses. Ann. NY. Acad. Sci. **1169**, 58–73. (10.1111/j.1749-6632.2009.04579.x) 19673755

[29] Kulashekhar S, Pekkola J, Palva JM, Palva S. 2016 The role of cortical beta oscillations in time estimation. Hum. Brain Mapp. **37**, 3262–3281. (10.1002/hbm.23239) 27168123 PMC6867325

[30] Saleh M, Reimer J, Penn R, Ojakangas CL, Hatsopoulos NG. 2010 Fast and slow oscillations in human primary motor cortex predict oncoming behaviorally relevant cues. Neuron **65**, 461–471. (10.1016/j.neuron.2010.02.001) 20188651 PMC3199221

[31] Zalta A, Large EW, Schön D, Morillon B. 2024 Neural dynamics of predictive timing and motor engagement in music listening. Sci. Adv. **10**, eadi2525. (10.1126/sciadv.adi2525) 38446888 PMC10917349

[32] Morillon B, Arnal LH, Schroeder CE, Keitel A. 2019 Prominence of delta oscillatory rhythms in the motor cortex and their relevance for auditory and speech perception. Neurosci. Biobehav. Rev. **107**, 136–142. (10.1016/j.neubiorev.2019.09.012) 31518638

[33] Schubotz RI. 2007 Prediction of external events with our motor system: towards a new framework. Trends Cogn. Sci. **11**, 211–218. (10.1016/j.tics.2007.02.006) 17383218

[34] Manning F, Schutz M. 2013 ‘Moving to the beat’ improves timing perception. Psychon. Bull. Rev. **20**, 1133–1139. (10.3758/s13423-013-0439-7) 23670284

[35] Morillon B, Schroeder CE, Wyart V. 2014 Motor contributions to the temporal precision of auditory attention. Nat. Commun. **5**, 5255. (10.1038/ncomms6255) 25314898 PMC4199392

[36] Schmidt-Kassow M, Heinemann LV, Abel C, Kaiser J. 2013 Auditory-motor synchronization facilitates attention allocation. Neuroimage **82**, 101–106. (10.1016/j.neuroimage.2013.05.111) 23732882

[37] Su YH, Pöppel E. 2012 Body movement enhances the extraction of temporal structures in auditory sequences. Psychol. Res. **76**, 373–382. (10.1007/s00426-011-0346-3) 21695472

[38] Ding N, Patel AD, Chen L, Butler H, Luo C, Poeppel D. 2017 Temporal modulations in speech and music. Neurosci. Biobehav. Rev. **81**, 181–187. (10.1016/j.neubiorev.2017.02.011) 28212857

[39] Nolan F, Jeon HS. 2014 Speech rhythm: a metaphor? Phil. Trans. R. Soc. B **369**, 20130396. (10.1098/rstb.2013.0396) 25385774 PMC4240963

[40] Elliott TM, Theunissen FE. 2009 The modulation transfer function for speech intelligibility. PLoS Comput. Biol. **5**, e1000302. (10.1371/journal.pcbi.1000302) 19266016 PMC2639724

[41] Giraud AL, Poeppel D. 2012 Cortical oscillations and speech processing: emerging computational principles and operations. Nat. Neurosci. **15**, 511–517. (10.1038/nn.3063) 22426255 PMC4461038

[42] Meyer L. 2018 The neural oscillations of speech processing and language comprehension: state of the art and emerging mechanisms. Eur. J. Neurosci. **48**, 2609–2621. (10.1111/ejn.13748) 29055058

[43] Poeppel D, Assaneo MF. 2020 Speech rhythms and their neural foundations. Nat. Rev. Neurosci. **21**, 322–334. (10.1038/s41583-020-0304-4) 32376899

[44] Cason N, Schön D. 2012 Rhythmic priming enhances the phonological processing of speech. Neuropsychologia **50**, 2652–2658. (10.1016/j.neuropsychologia.2012.07.018) 22828660

[45] Cason N, Astésano C, Schön D. 2015 Bridging music and speech rhythm: rhythmic priming and audio–motor training affect speech perception. Acta Psychol. **155**, 43–50. (10.1016/j.actpsy.2014.12.002) 25553343

[46] Berthault E, Chen S, Falk S, Morillon B, Schön D. 2024 Auditory and motor priming of metric structure improves understanding of degraded speech. Cognition **248**, 105793. (10.1016/j.cognition.2024.105793) 38636164

[47] Cason N, Hidalgo C, Isoard F, Roman S, Schön D. 2015 Rhythmic priming enhances speech production abilities: evidence from prelingually deaf children. Neuropsychology **29**, 102–107. (10.1037/neu0000115) 25068663

[48] Falk S, Dalla Bella S. 2016 It is better when expected: aligning speech and motor rhythms enhances verbal processing. Lang. Cogn. Neurosci. **31**, 699–708. (10.1080/23273798.2016.1144892)

[49] Falk S, Volpi-Moncorger C, Dalla Bella S. 2017 Auditory-motor rhythms and speech processing in French and German listeners. Front. Psychol. **8**, 395. (10.3389/fpsyg.2017.00395) 28443036 PMC5387104

[50] Assaneo MF, Rimmele JM, Sanz Perl Y, Poeppel D. 2021 Speaking rhythmically can shape hearing. Nat. Hum. Behav. **5**, 71–82. (10.1038/s41562-020-00962-0) 33046860

[51] Lubinus C, Keitel A, Obleser J, Poeppel D, Rimmele JM. 2023 Explaining flexible continuous speech comprehension from individual motor rhythms. Proc. R. Soc. B **290**, 20222410. (10.1098/rspb.2022.2410) PMC9975658 36855868

[52] Canette LH, Bedoin N, Lalitte P, Bigand E, Tillmann B. 2019 The regularity of rhythmic primes influences syntax processing in adults. Audit. Percept. Cogn. **2**, 163–179. (10.1080/25742442.2020.1752080)

[53] Fiveash A, Bedoin N, Lalitte P, Tillmann B. 2020 Rhythmic priming of grammaticality judgments in children: duration matters. J. Exp. Child Psychol. **197**, 104885. (10.1016/j.jecp.2020.104885) 32559634

[54] Bedoin N, Brisseau L, Molinier P, Roch D, Tillmann B. 2016 Temporally regular musical primes facilitate subsequent syntax processing in children with specific language impairment. Front. Neurosci. **10**, 245. (10.3389/fnins.2016.00245) 27378833 PMC4913515

[55] Fiveash A, Ladányi E, Camici J, Chidiac K, Bush CT, Canette LH, Bedoin N, Gordon RL, Tillmann B. 2023 Regular rhythmic primes improve sentence repetition in children with developmental language disorder. NPJ Sci. Learn. **8**, 23. (10.1038/s41539-023-00170-1) 37429839 PMC10333339

[56] Przybylski L, Bedoin N, Krifi-Papoz S, Herbillon V, Roch D, Léculier L, Kotz SA, Tillmann B. 2013 Rhythmic auditory stimulation influences syntactic processing in children with developmental language disorders. Neuropsychology **27**, 121–131. (10.1037/a0031277) 23356600

[57] Pickering MJ, Garrod S. 2013 An integrated theory of language production and comprehension. Behav. Brain Sci. **36**, 329–347. (10.1017/s0140525x12001495) 23789620

[58] Klepp A, van Dijk H, Niccolai V, Schnitzler A, Biermann-Ruben K. 2019 Action verb processing specifically modulates motor behaviour and sensorimotor neuronal oscillations. Sci. Rep. **9**, 15985. (10.1038/s41598-019-52426-9) 31690784 PMC6831701

[59] Pulvermüller F, Lutzenberger W, Preissl H. 1999 Nouns and verbs in the intact brain: evidence from event-related potentials and high-frequency cortical responses. Cereb. Cortex **9**, 497–506. (10.1093/cercor/9.5.497) 10450894

[60] Pulvermüller F. 2018 Neural reuse of action perception circuits for language, concepts and communication. Prog. Neurobiol. **160**, 1–44. (10.1016/j.pneurobio.2017.07.001) 28734837

[61] Strijkers K, Costa A. 2016 The cortical dynamics of speaking: present shortcomings and future avenues. Lang. Cogn. Neurosci. **31**, 484–503. (10.1080/23273798.2015.1120878)

[62] Strijkers K, Costa A, Pulvermüller F. 2017 The cortical dynamics of speaking: lexical and phonological knowledge simultaneously recruit the frontal and temporal cortex within 200 ms. NeuroImage **163**, 206–219. (10.1016/j.neuroimage.2017.09.041) 28943413

[63] Boersma P, Weenink D. 2023 Praat: doing phonetics by computer (6.3.10) [Computer software]. See http://www.praat.org/.

[64] Astésano C. 2016 Prosodic characteristics of reference French. In Varieties of spoken French (eds S Detey, J Durand, B Laks, C Lyche), pp. 68–85. Oxford, UK: Oxford University Press.

[65] Jun SA, Fougeron C. 2000 A phonological model of French intonation. In Intonation: analysis, modelling and technology (ed. A Botinis), pp. 209–242. Amsterdam, The Netherlands: Kluwer Academic Publishers.

[66] Ito J, Mester A. 2009 The extended prosodic word. In Phonological domains: universals and deviations (eds J Grijzenhout, B Kabak), pp. 135–194. Berlin, Germany: De Gruyter Mouton.

[67] Peperkamp SA. 1997 Prosodic words. Thesis, Universiteit van Amsterdam, The Netherlands.

[68] Nespor M, Vogel I. 2007 Prosodic phonology. Berlin, Germany: De Gruyter Mouton.

[69] Fairs A, Strijkers K. 2021 Can we use the internet to study speech production? Yes we can! evidence contrasting online versus laboratory naming latencies and errors. PLoS One **16**, e0258908. (10.1371/journal.pone.0258908) 34679082 PMC8535377

[70] FindingFive Team. 2023 FindingFive: An online platform for creating, running, and managing your experiments. See https://www.findingfive.com.

[71] R Core Team. 2013 R: a language and environment for statistical computing. Vienna, Austria: R Foundation for Statistical Computing. See http://www.R-project.org/.

[72] Bates D, Mächler M, Bolker B, Walker S. 2015 Fitting linear mixed-effects models using lme4. J. Stat. Softw. **67**, 1–48. (10.18637/jss.v067.i01)

[73] Lenth R, Singmann H, Love J, Buerkner P, Herve M. 2019 R package ‘emmeans’: estimated marginal means, aka least-squares means. R repository. See https://cran. r-project. org/web/packages/emmeans/index. html.

[74] Townsend JT, Ashby FG. 1983 Stochastic modeling of elementary psychological processes. Cambridge, UK: Cambridge University Press.

[75] Whelan R. 2008 Effective analysis of reaction time data. Psychol. Rec. **58**, 475–482. (10.1007/bf03395630)

[76] George D, Mallery P. 2019 IBM spss statistics 26 step by step: a simple guide and reference. London, UK: Routledge. (10.4324/9780429056765)

[77] Varnet L, Ortiz-Barajas MC, Erra RG, Gervain J, Lorenzi C. 2017 A cross-linguistic study of speech modulation spectra. J. Acoust. Soc. Am. **142**, 1976–1989. (10.1121/1.5006179) 29092595

[78] Du Y, Buchsbaum BR, Grady CL, Alain C. 2014 Noise differentially impacts phoneme representations in the auditory and speech motor systems. Proc. Natl Acad. Sci. USA **111**, 7126–7131. (10.1073/pnas.1318738111) 24778251 PMC4024897

[79] Cannon JJ, Patel AD. 2021 How beat perception co-opts motor neurophysiology. Trends Cogn. Sci. **25**, 137–150. (10.1016/j.tics.2020.11.002) 33353800 PMC9440376

[80] Coull JT, Droit-Volet S. 2018 Explicit understanding of duration develops implicitly through action. Trends Cogn. Sci. **22**, 923–937. (10.1016/j.tics.2018.07.011) 30266151

[81] De Kock R, Gladhill KA, Ali MN, Joiner WM, Wiener M. 2021 How movements shape the perception of time. Trends Cogn. Sci. **25**, 950–963. (10.1016/j.tics.2021.08.002) 34531138 PMC9991018

[82] Robbe D. 2023 Lost in time: relocating the perception of duration outside the brain. Neurosci. Biobehav. Rev. **153**, 105312. (10.1016/j.neubiorev.2023.105312) 37467906

[83] Jones MR, Boltz M. 1989 Dynamic attending and responses to time. Psychol. Rev. **96**, 459–491. (10.1037//0033-295x.96.3.459) 2756068

[84] Jones MR. 1976 Time, our lost dimension: toward a new theory of perception, attention, and memory. Psychol. Rev. **83**, 323–355. (10.1037//0033-295x.83.5.323) 794904

[85] Large EW, Jones MR. 1999 The dynamics of attending: how people track time-varying events. Psychol. Rev. **106**, 119–159. (10.1037//0033-295x.106.1.119)

[86] Rimmele JM, Morillon B, Poeppel D, Arnal LH. 2018 Proactive sensing of periodic and aperiodic auditory patterns. Trends Cogn. Sci. **22**, 870–882. (10.1016/j.tics.2018.08.003) 30266147

[87] Giroud J, Trébuchon A, Mercier M, Davis MH, Morillon B. 2024 The human auditory cortex concurrently tracks syllabic and phonemic timescales via acoustic spectral flux. Sci. Adv. **10**, eado8915. (10.1126/sciadv.ado8915) 39705351 PMC11661434

[88] Lidji P, Palmer C, Peretz I, Morningstar M. 2011 Listeners feel the beat: entrainment to English and French speech rhythms. Psychon. Bull. Rev. **18**, 1035–1041. (10.3758/s13423-011-0163-0) 21912999 PMC3219863

[89] Chalas N, Meyer L, Lo CW, Park H, Kluger DS, Abbasi O, Kayser C, Nitsch R, Gross J. 2024 Dissociating prosodic from syntactic delta activity during natural speech comprehension. Curr. Biol. **34**, 3537–3549.(10.1016/j.cub.2024.06.072) 39047734

[90] Inbar M, Grossman E, Landau AN. 2020 Sequences of intonation units form a ~ 1 Hz rhythm. Sci. Rep. **10**, 15846. (10.1038/s41598-020-72739-4) 32985572 PMC7522717

[91] Strauß A, Schwartz JL. 2017 The syllable in the light of motor skills and neural oscillations. Lang. Cogn. Neurosci. **32**, 562–569. (10.1080/23273798.2016.1253852)

[92] Mitchel AD, Weiss DJ. 2014 Visual speech segmentation: using facial cues to locate word boundaries in continuous speech. Lang. Cogn. Process. **29**, 771–780. (10.1080/01690965.2013.791703) 25018577 PMC4091796

[93] Sell AJ, Kaschak MP. 2009 Does visual speech information affect word segmentation? Mem. Cogn. **37**, 889–894. (10.3758/mc.37.6.889) PMC4606934 19679867

[94] Ogane R, Schwartz JL, Ito T. 2020 Orofacial somatosensory inputs modulate word segmentation in lexical decision. Cognition **197**, 104163. (10.1016/j.cognition.2019.104163) 31891832

[95] Abercrombie D. 1967 Elements of general phonetics. Edinburgh, UK: Edinburgh University Press.

[96] Pike KL. 1945 The intonation of American English. Ann Arbor, MI: University of Michigan Press.

[97] Delais-Roussarie E. 2022 Prosodic structure revisited: the need to disentangle rhythm from intonation. Rom. Jahrb. **73**, 31–69. (10.1515/roja-2022-0002)

[98] Rossi M. 1980 Le Français, langue sans accent? Stud. Phon. Montr. **15**, 13–51.

[99] Content A, Kearns RK, Frauenfelder UH. 2001 Boundaries versus onsets in syllabic segmentation. J. Mem. Lang. **45**, 177–199. (10.1006/jmla.2000.2775)

[100] Cutler A, Mehler J, Norris D, Segui J. 1986 The syllable’s differing role in the segmentation of French and English. J. Mem. Lang. **25**, 385–400. (10.1016/0749-596x(86)90033-1)

[101] Mehler J, Dommergues JY, Frauenfelder U, Segui J. 1981 The syllable’s role in speech segmentation. J. Verbal Learn. Verbal Behav. **20**, 298–305. (10.1016/s0022-5371(81)90450-3)

[102] Ordin M, Polyanskaya L, Gómez DM, Samuel AG. 2019 The role of native language and the fundamental design of the auditory system in detecting rhythm changes. J. Speech Lang. Hear. Res. **62**, 835–852. (10.1044/2018_jslhr-s-18-0299) 30969888

[103] Barchet AV, Henry MJ, Pelofi C, Rimmele JM. 2024 Auditory-motor synchronization and perception suggest partially distinct time scales in speech and music. Commun. Psychol. **2**, 2. (10.1038/s44271-023-00053-6) 39242963 PMC11332030

[104] Berent I, Platt M, Theodore R, Balaban E, Fried PJ, Pascual-Leone A. 2020 Speech perception triggers articulatory action: Evidence from mechanical stimulation. Front. Commun. **5**, 34. (10.3389/fcomm.2020.00034)

[105] Mares C, Echavarría Solana R, Assaneo MF. 2023 Auditory-motor synchronization varies among individuals and is critically shaped by acoustic features. Commun. Biol. **6**, 658. (10.1038/s42003-023-04976-y) 37344562 PMC10284880

[106] Pickering MJ, Garrod S. 2021 Understanding dialogue: language use and social interaction. Cambridge, UK: Cambridge University Press. (10.1017/9781108610728)

[107] Cheung C, Hamilton LS, Johnson K, Chang EF. 2016 The auditory representation of speech sounds in human motor cortex. eLife **5**, 12577. (10.7554/elife.12577) PMC4786411 26943778

[108] Leone C, Feys P, Moumdjian L, D’Amico E, Zappia M, Patti F. 2017 Cognitive-motor dual-task interference: a systematic review of neural correlates. Neurosci. Biobehav. Rev. **75**, 348–360. (10.1016/j.neubiorev.2017.01.010) 28104413

[109] Fiveash A, Bella SD, Bigand E, Gordon RL, Tillmann B. 2022 You got rhythm, or more: The multidimensionality of rhythmic abilities. Atten. Percept. Psychophys. **84**, 1370–1392. (10.3758/s13414-022-02487-2) 35437703 PMC9614186

[110] Flaugnacco E, Lopez L, Terribili C, Montico M, Zoia S, Schön D. 2015 Music training increases phonological awareness and reading skills in developmental dyslexia: a randomized control trial. PLoS One **10**, e0138715. (10.1371/journal.pone.0138715) 26407242 PMC4583182

[111] Pesnot Lerousseau J, Schön D. 2021 Musical expertise is associated with improved neural statistical learning in the auditory domain. Cereb. Cortex **31**, 4877–4890. (10.1093/cercor/bhab128) 34013316

[112] Schön D, Tillmann B. 2015 Short- and long-term rhythmic interventions: perspectives for language rehabilitation. Ann. NY. Acad. Sci. **1337**, 32–39. (10.1111/nyas.12635) 25773614

[113] Assaneo MF, Ripollés P, Orpella J, Lin WM, de Diego-Balaguer R, Poeppel D. 2019 Spontaneous synchronization to speech reveals neural mechanisms facilitating language learning. Nat. Neurosci. **22**, 627–632. (10.1038/s41593-019-0353-z) 30833700 PMC6435400

[114] Kern P, Assaneo MF, Endres D, Poeppel D, Rimmele JM. 2021 Preferred auditory temporal processing regimes and auditory-motor synchronization. Psychon. Bull. Rev. **28**, 1860–1873. (10.3758/s13423-021-01933-w) 34100222 PMC8642338

[115] Chandrasekaran C, Trubanova A, Stillittano S, Caplier A, Ghazanfar AA. 2009 The natural statistics of audiovisual speech. PLoS Comput. Biol. **5**, e1000436. (10.1371/journal.pcbi.1000436) 19609344 PMC2700967

[116] te Rietmolen N, Strijkers K, Morillon B. 2025 MotorSpeech: code and data. See https://github.com/DCP-INS/MotorSpeech. 10.1098/rspb.2025.0354 PMC11978457 40199360

[117] te Rietmolen N, Strijkers K, Morillon B. 2025 Supplementary material from: Moving rhythmically can facilitate naturalistic speech perception in a noisy environment. Figshare (10.6084/m9.figshare.c.7731452) PMC11978457 40199360

